# Integrated single-cell and bulk transcriptomic analyses reveal cDC1-centered ubiquitination dysregulation and identify UBE2F as a critical regulator in sepsis

**DOI:** 10.3389/fimmu.2026.1805849

**Published:** 2026-04-22

**Authors:** Cheng Li, Yiman Han, Zenglu Zheng, Chengya Huang, Zhiyun Liu, Jianwen Zhou, Rui Yang, Jingxiang Wu

**Affiliations:** 1 Department of Anesthesiology, Shanghai Chest Hospital, Shanghai Jiao Tong University School of Medicine, Shanghai, China; 2 Department of Anesthesiology, Second Affiliated Hospital of Naval Medical University (Shanghai Changzheng Hospital), Shanghai, China; 3 Department of Anesthesiology, No. 908th Hospital of the Chinese PLA Joint Logistic Support Force, Nanchang, China

**Keywords:** dendritic cells, sepsis, single-cell RNA sequencing, UBE2F, ubiquitination

## Abstract

**Background:**

Sepsis is a life-threatening syndrome with dysregulated immune responses and multiple organ dysfunction. However, precise diagnostic biomarkers and effective therapeutic targets for this syndrome are still lacking. Protein ubiquitination modulates inflammatory regulation and immune cell function, but the specific immune cell subsets that drive ubiquitination-associated immune dysregulation in human sepsis have not been clearly identified.

**Methods:**

An integrated analysis was performed using 315,220 single cells from two single-cell RNA sequencing (scRNA-seq) datasets in conjunction with independent bulk transcriptomic cohorts. We quantified cell-type responsiveness using Augur, inferred intercellular communication via CellChat, and identified ubiquitination-related gene networks through weighted gene co-expression network analysis (WGCNA) and subsequent multi-algorithm feature selection. Functional validation was conducted with lipopolysaccharide (LPS)-stimulated murine dendritic cells (DCs) line - DC2.4 *in vitro* and a cecal ligation and puncture (CLP) mouse model *in vivo*.

**Results:**

conventional Dendritic cells (cDCs) were identified as the most transcriptionally perturbed immune population in sepsis, with subsequent subclustering revealing that the type-1 conventional dendritic cells (cDC1) subset specifically exhibited pronounced activation of ubiquitination signatures. Cell-cell communication analysis identified TNF signaling as a sepsis-specific pathway, in which cDC1 functions as a critical mediator predominantly via the TNF–TNFRSF1B axis. Four ubiquitination-related genes (CUL1, UBE2F, UBE2N and UBE3A) demonstrated reproducible diagnostic performance across three independent cohorts. Notably, UBE2F showed the strongest upregulation and functional relevance in sepsis models. Both *in vitro* and *in vivo* experiments showed that silencing UBE2F markedly suppressed dendritic cell activation, decreased proinflammatory cytokine production and organ injury, and ultimately improved survival in septic mice.

**Conclusions:**

Our results reveal cDC1 as a key immune cell subset involved in ubiquitination-mediated immune dysregulation in sepsis and suggests that UBE2F may serve as a potential diagnostic biomarker and therapeutic target.

## 1 Introduction

Sepsis, a life-threatening syndrome driven by infection and characterized by dysregulated host responses and acute organ dysfunction, is commonly assessed using the Sequential Organ Failure Assessment (SOFA) score to determine disease severity and predict clinical outcomes (1). Despite advances in critical care, the global incidence of sepsis remains high, with in-hospital mortality showing only slight reductions, emphasizing the enduring impact of this condition (2). The pathophysiology of sepsis is highly intricate, involving significant disruptions in both innate and adaptive immunity, myeloid-cells dysfunction contributing to acquired immunosuppression, and extensive damage across various organ systems (3, 4). Standardized interventions (e.g., the SEP-1 sepsis bundle) and emerging pharmacological strategies (e.g., short-acting β-blockers) have shown efficacy in specific clinical settings (5, 6), the incorporation of biomarkers like high-sensitivity cardiac troponin (hs-cTn) and presepsin into clinical evaluations is on the rise (7–9), Nevertheless, mortality rates among critically ill sepsis patients remain unacceptably elevated. A more detailed molecular and cellular understanding of sepsis is thus imperative to facilitate precise diagnostics and targeted therapeutic interventions.

Ubiquitination is a highly conserved post-translational modification that regulates protein stability, localization and activity through the ubiquitin–proteasome system (UPS) (10). This process is coordinated by E1 activating enzymes, E2 conjugating enzymes, E3 ligases and the 26S proteasome, and is counterbalanced by deubiquitinating enzymes (DUBs) (11). Beyond proteostasis, ubiquitination modulates the crosstalk between ferroptosis and autophagy, and preserves the structural integrity of the UPS (12, 13). Increasing evidence links ubiquitin-dependent mechanisms to a diverse array of biological and pathological processes, including tumor progression (14), epigenomic stability (15, 16) and the regulation of cardiac fibrosis. In sepsis, aberrant ubiquitination can modulate inflammatory signaling via non-degradative ubiquitin chains (17), reshape immune-cell function (18), and regulate cell death and autophagy, thereby contributing to sepsis-induced injury in the heart, lung, liver, kidney and gut (19, 20). Nevertheless, the pivotal regulators of ubiquitination, the specific immune cell subsets involved, and the regulatory frameworks linking ubiquitination to inflammatory restructuring in sepsis remain incompletely characterized, hampering translational endeavors aimed at targeting ubiquitin pathways.

The rapid advancement of single-cell and bulk transcriptomic technologies has facilitated detailed analysis of immune-cell diversity and offers complementary insights into sepsis biology (21). scRNA-seq has been utilized to uncover ubiquitination-related regulatory mechanisms in diseases like pancreatic cancer (22, 23), while bulk RNA-seq remains a robust and widely used approach for identifying diagnostic and prognostic biomarkers in sepsis (24, 25). However, there is no comprehensive framework available to identify key ubiquitination regulators and the specific immune cell subsets responsible for ubiquitination-related dysregulation in sepsis.

In this study, we employed a multi-scale integrative framework combining single-cell and bulk transcriptomics with network biology to map the sepsis-associated ubiquitinome. We identified cDC1 as the most transcriptionally perturbed subset, with aberrant ubiquitination activity induced by a sepsis-specific TNF–TNFRSF1B signaling axis. Through further integration of bulk transcriptomic data using multidimensional bioinformatic techniques and machine-learning algorithms, we pinpointed four key ubiquitination-related genes—CUL1, UBE2F, UBE2N, and UBE3A—as central regulators with substantial diagnostic relevance in sepsis. Notably, experimental validation highlighted UBE2F as a pivotal effector in modulating DCs activation, inflammatory responses, and organ damage in sepsis models. These results establish a ubiquitination-centered regulatory axis within cDC1 in sepsis, offering a novel mechanistic rationale for precision diagnostic and therapeutic interventions.

## 2 Materials and methods

### 2.1 Public data acquisition

All datasets analyzed in this study were obtained from the NCBI Gene Expression Omnibus (GEO) database. scRNA-seq datasets included GSE167363, comprising peripheral blood mononuclear cells (PBMCs) from 10 patients with sepsis and 2 healthy controls, and GSE220189, including PBMCs from 21 patients with sepsis and 23 healthy controls. Bulk transcriptomic datasets consisted of GSE69528 (83 sepsis patients and 28 healthy controls) and GSE13015, which contains two microarray platforms: GPL6106 (48 sepsis patients and 19 healthy controls) and GPL6947 (29 sepsis patients and 10 healthy controls). A curated set of ubiquitination-related genes (URGs) was obtained from a previous research (26) and the ESBL database (https://esbl.nhlbi.nih.gov/Databases/KSBP2/Targets/Lists/E3-ligases/).

### 2.2 scRNA−seq data processing, quality control and cell annotation

Raw scRNA-seq data were subjected to initial quality control. Subsequent preprocessing was performed using Scanpy (v1.9.3), including total-count normalization (pp.normalize_total), log-transformation (pp.log1p), identification of 2,000 highly variable genes, and data scaling (pp.scale). To integrate samples across datasets and mitigate batch effects, a variational inference model was implemented using scVI (v1.2.1). Based on the scVI latent representation, a neighborhood graph was constructed with pp.neighbors (n_neighbors = 10), followed by clustering using the Leiden algorithm (resolution = 0.6). Uniform Manifold Approximation and Projection (UMAP) was applied for dimensionality reduction and visualization. Cell-type annotation was performed by integrating canonical marker genes with predictions from the scType tool, followed by manual curation to refine key immune cell subpopulations.

### 2.3 Identification of sepsis−responsive cell subpopulations using augur

To quantify the responsiveness of individual cell types to the sepsis-associated perturbation, Augur analysis was conducted using the pertpy framework (27). Augur computed cell-type–specific classification performance by distinguishing sepsis from control states, using a random forest classifier with a subsample size of 10 cells. For each cell type, repeated subsampling was performed, and the mean receiver operating characteristic area under the curve (ROC-AUC) across iterations was calculated. Only cell types with cell numbers equal to or exceeding the subsample size were retained. Cell populations were ranked according to their Augur scores to prioritize sepsis-responsive subpopulations.

### 2.4 Subclustering of key cell subpopulations and AUCell scoring

Cell subpopulations identified as highly responsive by Augur were further subjected to subclustering using the Leiden algorithm with a lower resolution (resolution = 0.1) to resolve finer cellular heterogeneity. To quantify ubiquitination-related transcriptional activity, AUCell analysis was performed to calculate per-cell AUC scores (28) based on URGs expression. Differences in URGs activity across conditions and subclusters were visualized using stacked violin plots.

### 2.5 CellChat cell–cell communication analysis

Intercellular communication networks were inferred using CellChat (v1.6.1). A CellChat object was constructed from the normalized single-cell expression matrix. Highly expressed genes and candidate ligand–receptor interactions were identified using identifyOverExpressedGenes and identifyOverExpressedInteractions, respectively (29). Communication probabilities were estimated with computeCommunProb (nboot = 100), and pathway-level communication strength was summarized using aggregateNet. Analyses focused on TNF signaling pathways, with netAnalysis_signalingRole used to characterize sender, receiver, mediator and influencer roles, and netAnalysis_contribution applied to quantify the contribution of key ligand–receptor pairs.

### 2.6 Identification of sepsis−related URGs, WGCNA and functional enrichment analyses

Differential expression analysis of GSE69528 was performed using the limma package (v3.58.1), with thresholds set at |log_2_ fold change| > 1 and adjusted P value < 0.05 to identify differentially expressed genes (DEGs) between sepsis and control samples (30). Before differential expression analysis, sample-level quality control was performed using low-dimensional visualization (UMAP) based on the global gene-expression matrix. Three samples that were clearly separated from the main sample cluster were considered outliers and excluded from subsequent analyses. The GSE13015 datasets (GPL6106 and GPL6947) were normalized and reserved for downstream validation. WGCNA (31) was conducted to identify gene modules associated with the sepsis phenotype, with a soft-thresholding power of β = 9 selected to achieve scale-free topology (R² > 0.85). Module–trait correlations were calculated using module eigengenes, and key modules were selected based on their association with sepsis. Sepsis-related URGs were defined as the intersection of genes from key WGCNA modules, DEGs from GSE69528, and the predefined URGs list. Functional enrichment analyses were performed using clusterProfiler (v4.10.0), including Gene Ontology (BP, CC, MF) and KEGG pathway over-representation analyses (q value < 0.05). GSEA was conducted using KEGG gene sets with 1,000 permutations and false discovery rate correction. Reactome pathway analysis was performed using ReactomePA (v1.46.0).

### 2.7 pySCENIC transcription factor analysis

Transcription factor (TF) regulatory networks were reconstructed for key cell subpopulations using pySCENIC (32). GRNBoost2 and GENIE3 algorithms were employed to infer TF–target relationships, followed by motif enrichment and transcription factor binding site validation to refine regulons. The minimum number of genes per module/regulon was set to 20 (min_genes = 20). Motif enrichment was performed using rank_threshold = 5000, auc_threshold = 0.05, and NES threshold = 3.0, with motif annotation filters of min_orthologous_identity = 0.0 and max_similarity_fdr = 0.001. Regulon activity was quantified using AUCell, and regulon specificity scores (RSS) were calculated to identify the top five TFs for each subpopulation. KEGG enrichment analysis of TF target genes was performed to identify TFs associated with ubiquitination-related pathways. Protein–protein interaction (PPI) networks for core TF target genes were constructed using the STRING database with a confidence score > 0.7.

### 2.8 Machine−learning–based selection of key genes

Ubiquitination-related target genes regulated by core TFs were further screened using three machine-learning approaches. The analyses were performed based on the candidate-gene expression matrix derived from the bulk transcriptomic cohort. Before model training, gene expression values were standardized to reduce the influence of differences in feature scale. Random forest models were constructed using the randomForest package, and genes with MeanDecreaseGini values above the median were retained. Least absolute shrinkage and selection operator (LASSO) regression was implemented using the glmnet package, with ten-fold cross-validation used to determine the optimal penalty parameter (λ); genes with non-zero coefficients were selected (31). Support vector machine–recursive feature elimination (SVM-RFE) with a linear kernel was applied to rank features and identify the optimal gene subset. Candidate subsets were evaluated by cross-validation, and the subset with the highest cross-validated accuracy was selected. In the present study, the best performance was achieved with 8 variables (accuracy = 0.905, kappa = 0.728), and this 8-gene set was therefore defined as the optimal SVM-RFE subset. The intersection of genes identified by all three algorithms was defined as the final set of candidate genes.

### 2.9 Clinical relevance assessment of candidate genes

The diagnostic performance of candidate genes was evaluated by ROC analysis using the pROC package (33) across three independent datasets: GSE69528, GSE13015_GPL6106 and GSE13015_GPL6947. AUC values and 95% confidence intervals were calculated, with AUC > 0.70 considered indicative of satisfactory diagnostic performance. Gene expression differences between sepsis and control groups were visualized using boxplots generated with ggplot2, and statistical significance was assessed using Wilcoxon rank-sum tests. In single-cell datasets, dot plots were used to display expression patterns across cell types and subclusters.

### 2.10 Acquisition of DCs line and generation of BMDCs

The murine DCs line - DC2.4 was obtained from a commercial source (Merck Millipore). DC2.4 cells were cultured in RPMI 1640 medium supplemented with 10% fetal bovine serum (FBS) and 1% penicillin–streptomycin at 37 °C in a humidified incubator with 5% CO_2_ (34). Cells were routinely passaged at 70–80% confluence using 0.25% trypsin–EDTA, and only low-passage cells were used for all experiments.

Bone marrow–derived dendritic cells (BMDCs) were generated from C57BL/6 mice as previously described with minor modifications (35). Briefly, bone marrow cells were flushed from femurs and tibias under sterile conditions and cultured in RPMI 1640 medium supplemented with 10% FBS, 1% penicillin–streptomycin, recombinant murine GM-CSF, and IL-4. Cells were maintained at 37 °C in a humidified incubator with 5% CO_2_, and half of the medium was replaced every 2–3 days. On day 7, non-adherent and loosely adherent cells displaying typical dendritic cell morphology were harvested for subsequent experiments.

### 2.11 Cell transfection

For gene modulation, DCs and BMDCs were transfected with the indicated small interfering RNAs (siRNAs) using Lipofectamine 3000 (Thermo Fisher Scientific) according to the manufacturer’s protocol (36). Cells were seeded at an appropriate density and transfected in serum-free medium, which was replaced with complete culture medium after 6 h. Transfection efficiency was evaluated by western blot analysis. Cells transfected with empty vectors or scrambled siRNA sequences were used as negative controls.

### 2.12 Establishment of cells and animals models of sepsis

To establish an *in vitro* sepsis-like inflammatory model, DC2.4 cells were seeded at the indicated density and allowed to adhere overnight, followed by stimulation with lipopolysaccharide (LPS; Merck, L2880) at a final concentration of 200 ng/mL for the indicated time periods prior to downstream analyses.

Male C57BL/6 mice (6–8 weeks old) were purchased from Shanghai JieSiJie Laboratory Animals Company, LTD. Animals were housed in individually ventilated cages (IVCs) maintained at a controlled temperature of 22 ± 1 °C, following a consistent 12-hour circadian rhythm. All animal studies and experimental procedures were approved by the Animal Care and Use Committee of Shanghai Chest Hospital, Shanghai Jiao Tong University, on February 26, 2025 [permission no. KS(Y)25200].

All mice underwent a 12 h fasting period prior to the experiment. Surgical site sterilization and preparation were performed following anesthesia with 2% sodium pentobarbital 50mg/kg intraperitoneally for induction, and 1%~3% isoflurane inhalation for maintenance. The distal end of the ileocecal valve was subsequently detached and ligated at 1/3 of the cecum using 4–0 silk sutures. After puncturing the ligated end of the cecum with a 22-gauge needle and extracting a tiny amount of feces, 4–0 silk sutures were used to close the peritoneum and intermittently suture the skin (37). A subcutaneous injection of 50 ml/kg normal saline was administered to prevent shock. After recovering for 30 minutes at 37 °C on a warming plate, the mice were returned to their cages.

### 2.13 Hematoxylin and eosin staining

At 24 h after model induction, mice were euthanized following orbital blood collection. The right lung was harvested and immediately snap-frozen at −80 °C for subsequent molecular analyses, while the left lung was fixed in 4% paraformaldehyde for histological evaluation. Paraffin-embedded lung tissues were sectioned and stained with H&E according to standard protocols (38). Lung injury was independently assessed by an experienced investigator blinded to group allocation using a standardized five-point histopathological scoring system, based on alveolar wall thickness, inflammatory cell infiltration, hemorrhage and structural integrity.

### 2.14 RNA extraction and quantitative real−time PCR

Total RNA was extracted from cultured cells or tissue samples using a commercial RNA extraction kit (Beyotime, R0026) following the manufacturer’s instructions. RNA purity and concentration were assessed spectrophotometrically, and equal amounts of RNA were reverse-transcribed into complementary DNA (cDNA) under standard conditions. qRT-PCR was performed using gene-specific primers designed with NCBI Primer-BLAST (39). Amplification reactions were conducted in a total volume of 10 μL for 40 cycles with fluorescence detection. GAPDH was used as an internal reference gene, and relative gene expression levels were calculated using the 2^−ΔΔCt method. Primer sequences are provided in **Supplementary Table 1**.

### 2.15 Flow cytometry

Cells and mouse spleen tissues were collected and processed into single-cell suspensions, washed with cold PBS, and then incubated with fluorochrome-conjugated surface antibodies (CD80, Cat.104707, BioLegend; CD86, Cat.105007, BioLegend; MHC II, Cat.116421, BioLegend; and Annexin V, Cat.640907, BioLegend) in the dark at 4 °C for 30 min for subsequent flow cytometric analysis (40). Following staining, cells were washed, resuspended in staining buffer, and analyzed using a CytoFLEX flow cytometer (Beckman Coulter). Flow cytometry data were processed and analyzed using FlowJo software (v10). Appropriate isotype and fluorescence-minus-one controls were included to ensure accurate gating.

### 2.16 Enzyme−linked immunosorbent assay

Serum concentrations of tumor necrosis factor-α (TNF-α), interleukin-6 (IL-6) and interleukin-1β (IL-1β) were quantified using commercially available mouse ELISA kits (Beyotime Biological Technology, Shanghai, China) in accordance with the manufacturer’s protocols. Orbital blood samples were allowed to clot at room temperature for 30 min and centrifuged at 2,000 × g for 15 min at 4 °C. Serum was aliquoted and stored at −80 °C until analysis. Standards were serially diluted to generate standard curves, and all samples were assayed in duplicate (41). Optical density was measured at 450 nm with a reference wavelength of 630 nm. Cytokine concentrations were calculated using four-parameter logistic regression.

### 2.17 Western blot analysis

Tissue and cell lysates were prepared using RIPA lysis buffer (Beyotime, P0013B) supplemented with protease inhibitors. Protein concentrations were determined by bicinchoninic acid (BCA) assay (Thermo Fisher Scientific, 23225). Equal amounts of protein were separated by 10% SDS–PAGE and transferred onto polyvinylidene fluoride (PVDF) membranes (Millipore, IPVH00010). Membranes were blocked with 5% nonfat milk for 1 h at room temperature and incubated overnight at 4 °C with primary antibodies against UBE2F (Proteintech, 17056-1-AP), and GAPDH (Beyotime, AG019-1) (42). After incubation with HRP-conjugated secondary antibodies (Cell Signaling Technology, 7074/7076) for 1 h at room temperature, protein bands were visualized using enhanced chemiluminescence (ECL; Tanon, 180-5010). Band intensities were quantified using ImageJ software (v1.8.0) and normalized to GAPDH.

### 2.18 Statistical analysis

Single-cell and bulk transcriptomic analyses were conducted using Python (v3.9.16) and R (v4.3.3), respectively. Statistical analyses were performed using GraphPad Prism (v9.5.0) (43). Data are presented as mean ± SD from at least three independent experiments. Normality was assessed using the Shapiro–Wilk test. Comparisons between two groups were performed using an unpaired two-tailed Student’s t-test or the Mann–Whitney U test, as appropriate. For comparisons among three or more groups, we applied one-way or two-way ANOVA followed by Tukey’s or Sidak’s *post hoc* tests. Statistical significance was defined as P < 0.05.

## 3 Results

### 3.1 Integration of scRNA-seq datasets reveals immune landscape remodeling in human sepsis

**Figure 1:**
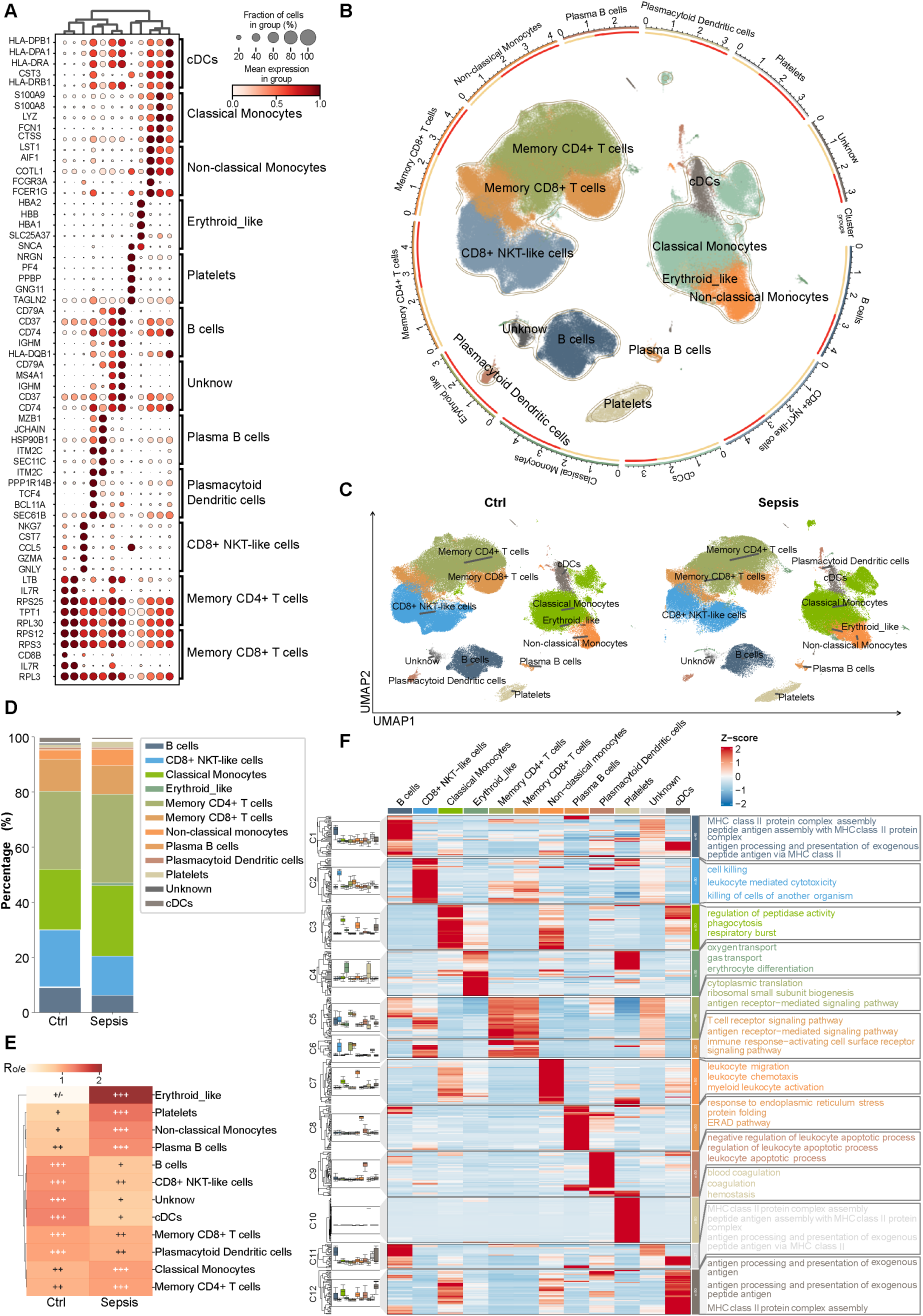
Integration of scRNA-seq datasets reveals immune landscape remodeling in human sepsis. **(A)** Marker gene expression for identified cell subpopulations. **(B)** Circular summary of cell-type annotation integrating marker expression and functional labels. **(C)** UMAP of Control (left) and Sepsis (right) samples. **(D)** Stacked bar plot of cell-type proportions in Control vs. Sepsis. **(E)** Ro/e enrichment heatmap by group. **(F)** Multi-omics heatmap integrating DEGs, GO-BP enrichment and violin plots to illustrate functional reprogramming across cell types.

We first integrated two human sepsis scRNA-seq datasets (GSE167363 and GSE220189), and obtained 315,220 high-quality peripheral blood mononuclear cells (PBMCs). Following batch correction through scVI and unsupervised clustering utilizing the Leiden algorithm (with a resolution of 0.6), technical batch effects were effectively mitigated, resulting in a clear resolution of cellular populations in UMAP space, delineating 22 unique clusters (**Supplementary Figure 1A**). By integrating canonical marker gene expression with automated annotation using scType, followed by manual curation (**Figure 1A**), we identified 11 major immune cell subpopulations, including B cells, CD8^+^ NKT-like cells, Classical Monocytes, Erythroid-like cells, Memory CD4^+^ T cells, Memory CD8^+^ T cells, Non-classical Monocytes, Plasma B cells, Plasmacytoid Dendritic cells, Platelets and cDCs. Clusters that could not be confidently assigned were designated as “Unknown” (**Figure 1B**). UMAP visualization illustrated significant differences in the spatial distribution of several immune cell populations between sepsis and control samples, particularly among platelets, non-classical monocytes, B cells and cDCs (**Figure 1C**). Quantitative analysis of cell-type proportions (stacked bar plots and Ro/e enrichment heatmaps) showed that cDCs and B cells were markedly reduced in sepsis, while non-classical monocytes were significantly expanded. This finding indicates substantial remodeling of immune composition under septic conditions (**Figures 1D, E**). To further characterize functional alterations across immune compartments, we conducted an integrated analysis of differentially expressed genes and GO-BP enrichment. This analysis revealed that cDCs and B cells were predominantly enriched for pathways related to antigen processing and presentation, while non-classical monocytes exhibited preferential enrichment for pathways associated with leukocyte activation and inflammatory responses. These results highlight extensive functional reprogramming across immune cell subpopulations during sepsis (**Figure 1F**).

### 3.2 cDC1 as a central cDCs subset driving ubiquitin-related immune remodeling in sepsis via TNF signaling

**Figure 2:**
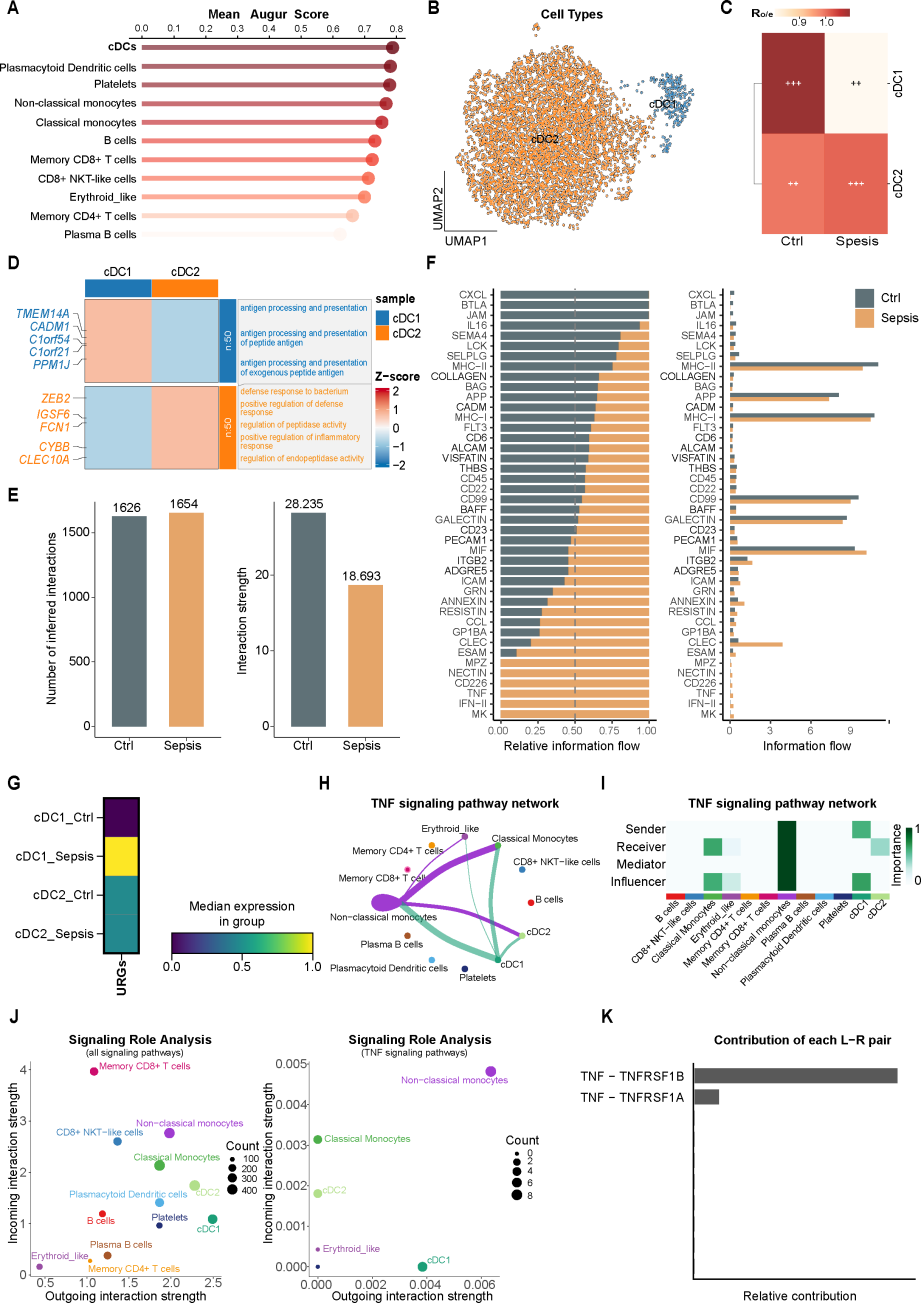
cDC1 as a central conventional dendritic cell subset driving ubiquitin-related immune remodeling in sepsis via TNF signaling. **(A)** Bar plot of mean Augur scores for subpopulations across cell types, indicating relative sensitivity to the sepsis perturbation. **(B)** UMAP showing the spatial distribution of cDC1 and cDC2 cells. **(C)** Ro/e heatmap of cDC1/cDC2 enrichment in Ctrl vs. Sepsis. **(D)** Functional enrichment heatmap of cDC1 vs. cDC2 DEGs with Z-score expression and sample grouping. **(E)** Quantitative comparison of the total number of inferred interactions (left) and overall interaction strength (right) between Control and Sepsis groups. **(F)** Information flow analysis identifying significant signaling pathways. The bar plots display the relative (left) and absolute (right) information flow, highlighting signaling pathways specifically enriched in Control (grey) or Sepsis (orange) conditions. **(G)** Heatmap of URGs expression across four groups (cDC1_Ctrl, cDC1_Sepsis, cDC2_Ctrl, cDC2_Sepsis). **(H)** TNF signaling cell–cell interaction network (nodes = cell types; edges = ligand–receptor interactions). **(I)** Heatmap of role importance (Sender/Receiver/Mediator/Influencer) in TNF network. **(J)** Signaling role analysis: all pathways (left) and TNF pathway only (right); point size corresponds to the number of interactions (count). **(K)** Relative contribution of TNF–TNFRSF1B and TNF–TNFRSF1A pairs to overall communication.

To identify immune cell populations most responsive to septic perturbation, we quantified cell-type responsiveness using Augur. Among all annotated immune populations, cDCs exhibited the highest mean Augur score (mean AUC = 0.787), indicating that they are the most transcriptionally responsive cell type in sepsis (**Figure 2A**). Based on this observation, we selected cDCs for further subclustering. Using the Leiden algorithm at a lower resolution (resolution = 0.1), two transcriptionally distinct cDCs subsets were identified and annotated as cDC1 and cDC2 according to canonical marker gene expression patterns (**Figure 2B**; **Supplmentary Figure S1B**). A subtype-specific Ro/e heatmap comparing control and sepsis samples revealed a significant reduction in the relative abundance of cDC1, accompanied by a concomitant increase in cDC2 during sepsis (**Figure 2C**). Functional enrichment analysis of subtype-specific differentially expressed genes demonstrated that cDC1 were primarily enriched for pathways related to antigen processing and presentation, whereas cDC2 exhibited preferential activation of inflammation- and host defense–associated pathways, including defense response to bacterium and positive regulation of inflammatory response (**Figure 2D**). To evaluate ubiquitination activity at single-cell resolution, we applied AUCell to calculate AUC scores for URGs. The URGs activity in cDC1 was significantly higher in septic samples than in control cDC1 and cDC2 subsets. These results suggested that cDC1 acts as a cellular hub for ubiquitination-modulated immune regulation in sepsis (**Figure 2G**).

To delineate the intercellular communication landscape involving cDC1, we performed ligand–receptor interaction analysis utilizing CellChat. Global analysis revealed substantial remodeling of intercellular communication networks in sepsis, with multiple signaling pathways exhibiting altered activity compared to controls (**Figure 2E**). Differential pathway analysis identified TNF signaling as a sepsis-specific activated pathway, displaying robust communication activity in sepsis but minimal engagement under control conditions (**Figure 2F**). Focusing on TNF-mediated interactions, cell-cell communication network analysis indicated that TNF signaling is initiated predominantly by non-classical monocytes, relayed through cDC1 as a central mediator, and ultimately received by monocytes and cDC2, establishing a regulatory axis (**Figure 2H**). Role-importance analysis further demonstrated that cDC1 occupy dominant Sender and Influencer roles within the TNF signaling network (**Figure 2I**). Consistently, cDC1 showed higher outgoing interaction strength than cDC2 within the TNF pathway (**Figure 2J**). Ligand–receptor contribution analysis identified TNF–TNFRSF1B as the principal ligand–receptor pair driving TNF pathway communication (**Figure 2K**). Collectively, these single-cell analyses indicate that cDC1 orchestrate ubiquitination-modulated immune remodeling in sepsis through TNF-centered intercellular signaling networks.

### 3.3 Bulk transcriptomic analysis and WGCNA identify ubiquitination-related gene modules associated with sepsis

**Figure 3:**
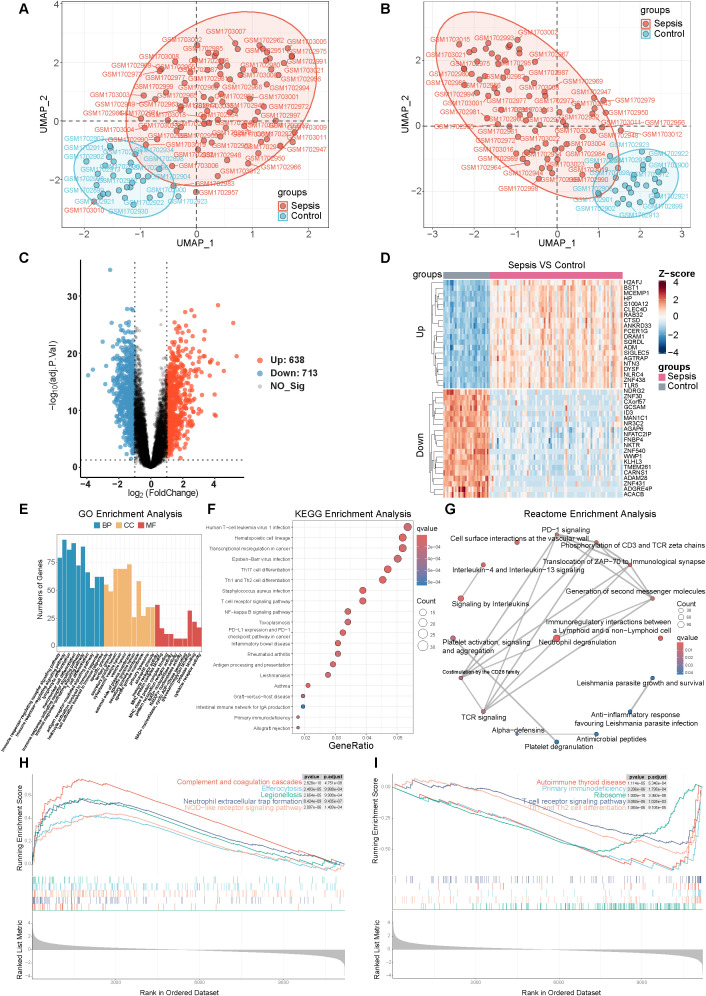
Bulk RNA-seq differential expression and functional enrichment analyses. **(A)** UMAP showing sample distribution (sepsis vs. control) before removal of outlier samples. **(B)** UMAP showing sample distribution after removal of outliers. **(C)** Volcano plot of DEGs (sepsis vs. control). **(D)** Z-score normalized heatmap of top 20 significantly dysregulated genes. **(E–I)** Enrichment analyses: GO (BP/CC/MF), KEGG, GSEA (top activated/suppressed pathways) and Reactome network.

To validate the single-cell findings at the cohort level and to identify gene networks associated with sepsis, we analyzed the bulk transcriptomic dataset GSE69528. Unsupervised UMAP-based clustering revealed three outlier samples, which were excluded to ensure the robustness of subsequent analyses (**Figures 3A, B**). Differential expression analysis conducted using limma identified 1,351 DEGs between sepsis and control samples, comprising 638 upregulated and 713 downregulated genes (**Figure 3C**). The top 20 most significantly dysregulated genes are presented in a Z-score–normalized heatmap (**Figure 3D**). Functional enrichment analysis of the DEGs demonstrated extensive immune dysregulation in sepsis. GO-BP analysis indicated significant enrichment of immune-related processes, including the regulation of immune response and immune cell activation (**Figure 3E**). Correspondingly, KEGG pathway analysis underscored the enrichment of NF-κB signaling, T cell receptor signaling, and antigen processing and presentation pathways (**Figure 3F**). Reactome analysis further identified neutrophil degranulation as a notably enriched pathway (**Figure 3G**). GSEA revealed that pathways associated with efferocytosis, neutrophil extracellular trap formation, and NOD-like receptor signaling were upregulated in sepsis, while adaptive immune pathways, such as primary immunodeficiency, T cell receptor signaling, and Th1/Th2 differentiation, were downregulated (**Figures 3H, I**). Collectively, these findings suggest a shift toward innate immune activation, accompanied by the suppression of adaptive immune programs in sepsis.

**Figure 4:**
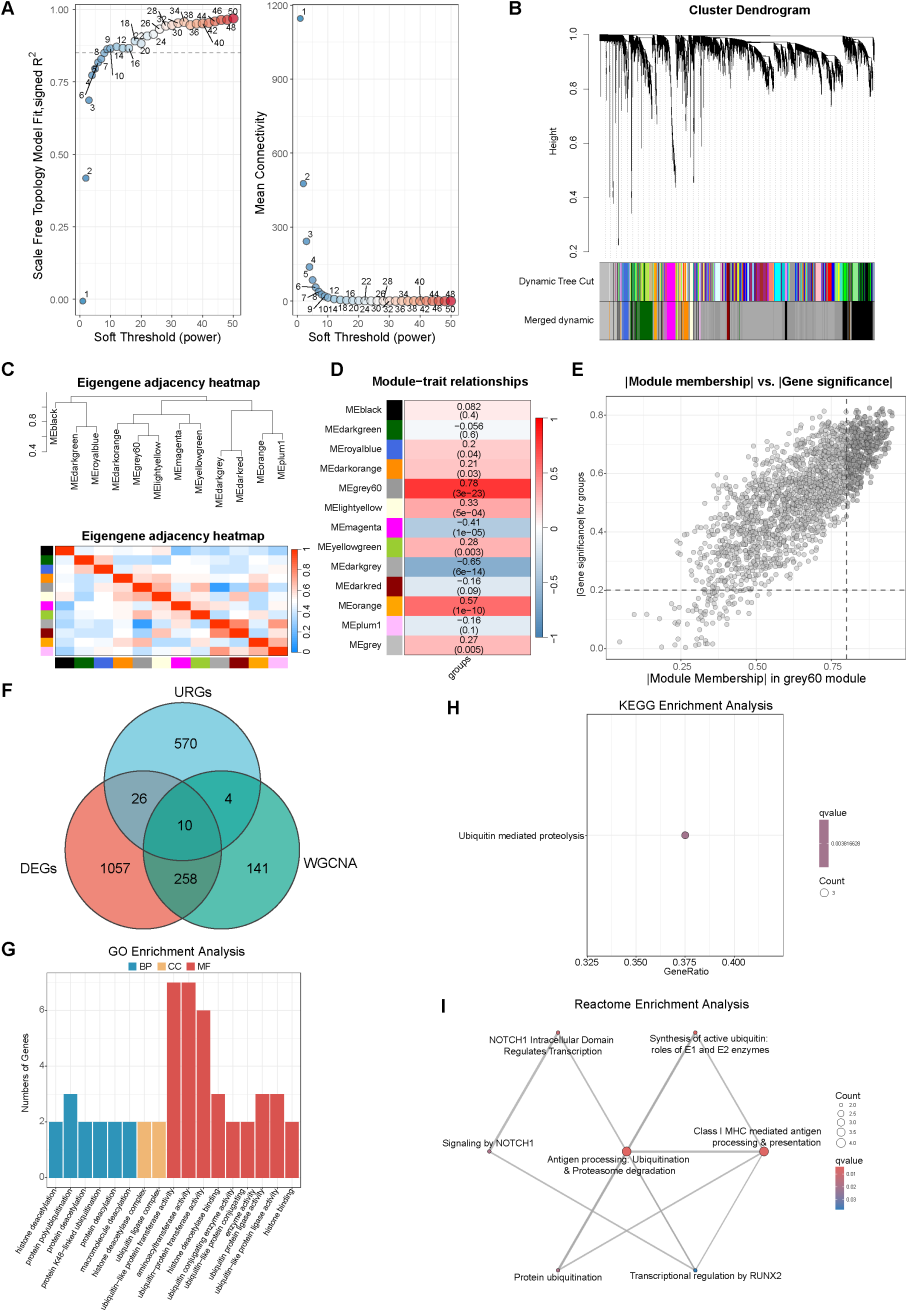
WGCNA of DEGs and enrichment of intersecting modules. **(A)** Soft-threshold selection for WGCNA. **(B)** Gene dendrogram with dynamic tree cut modules. **(C, D)** Module eigengene clustering and module–trait correlations. **(E)** Scatter plot of |Module Membership| versus |Gene Significance| for genes in the grey60 module. **(F)** Venn diagram of DEGs, URGs and WGCNA hub genes. **(G–I)** GO/KEGG/Reactome enrichment for intersecting genes.

To identify gene networks associated with the sepsis phenotype, we performed WGCNA on the DEGs. A soft-thresholding power of 9 was selected to achieve scale-free topology (R² > 0.85) (**Figure 4A**). Hierarchical clustering identified 12 distinct gene modules (**Figure 4B**), with clear module separation confirmed by the adjacency heatmap (**Figure 4C**). Module–trait correlation analysis revealed that the grey60 module exhibited the strongest positive association with sepsis (r = 0.78, P = 3 × 10^-^²³) (**Figure 4D**). Using thresholds of |Gene Significance| > 0.2 and |Module Membership| > 0.8, we identified 413 hub genes within this module (**Figure 4E**). Intersecting the grey60 hub genes with the DEGs and the predefined URGs set yielded ten sepsis-associated ubiquitination-related genes (**Figure 4F**). These genes were enriched for ubiquitin-related enzymatic processes in GO analysis (**Figure 4G**), ubiquitin-mediated proteolysis in KEGG analysis (**Figure 4H**), and antigen processing via ubiquitination and proteasome degradation in Reactome analysis (**Figure 4I**). These results further support a close association between coordinated ubiquitination-related gene networks and the sepsis phenotype.

### 3.4 pySCENIC and machine-learning analyses prioritize seven ubiquitin-related hub genes in cDC1

**Figure 5:**
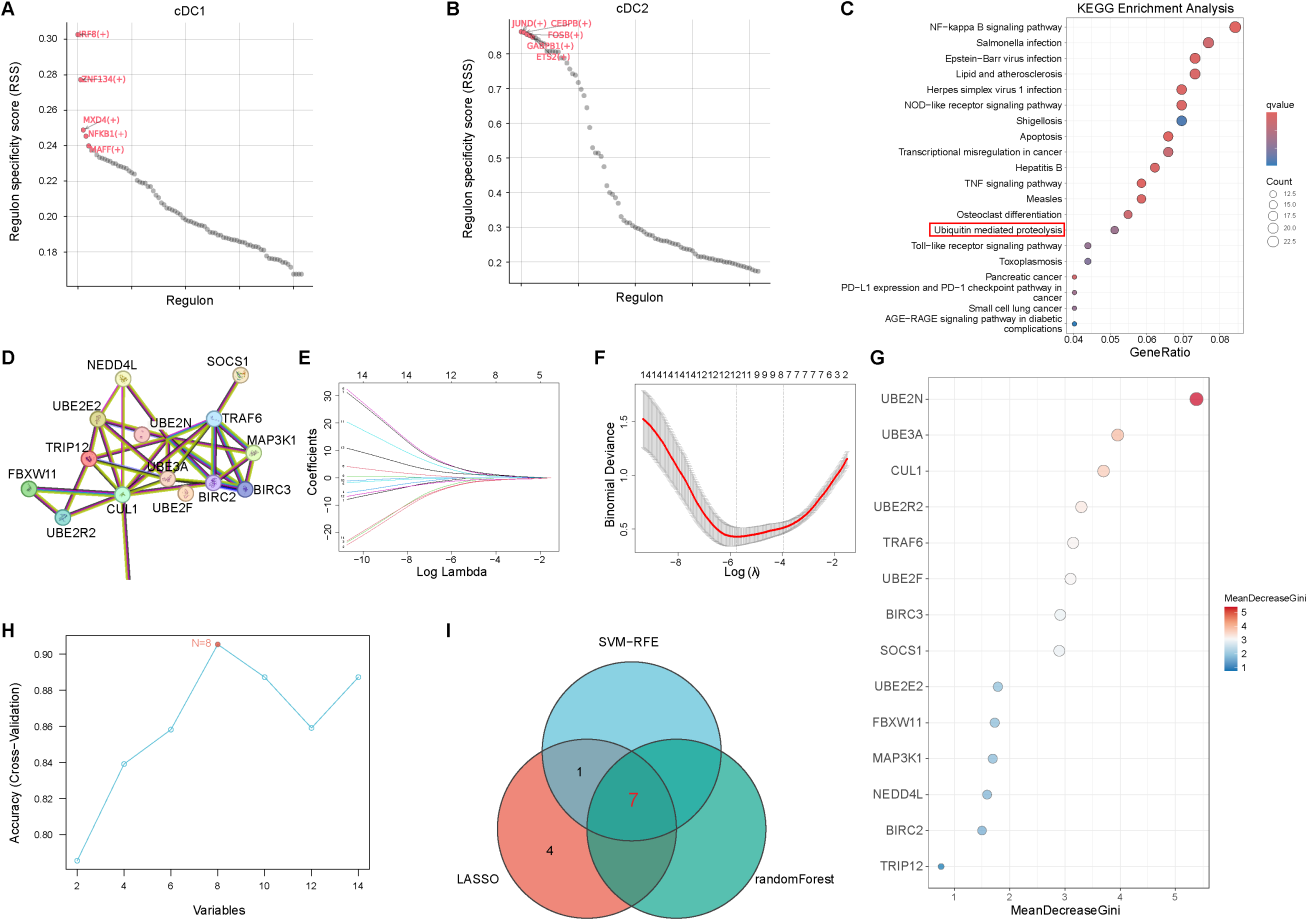
Transcriptional drivers and machine-learning prioritize seven ubiquitin-related hub genes in cDC1. **(A, B)** pySCENIC RSS distributions for cDC1 and cDC2. **(C)** KEGG enrichment bubble plot for NFKB1 target genes. **(D)** PPI network for NFKB1 target genes. **(E, F)** LASSO coefficient paths and cross-validation. **(G)** Gene importance ranking from the random forest model. **(H)** Cross-validation accuracy curve for SVM-RFE. **(I)** Venn diagram of genes selected by LASSO, SVM-RFE and random forest; seven consensus hub genes identified.

To elucidate the transcriptional regulatory mechanisms underlying elevated URGs activity in cDCs subsets, we reconstructed regulon networks using pySCENIC on cDC1 and cDC2 populations. RSS analysis identified distinct transcriptional regulators between the two subsets. The top five regulons in cDC1 were IRF8, ZNF134, MXD4, NFKB1 and MAFF, whereas cDC2 were characterized by JUND, CEBPB, FOSB, GABPB1 and ETS2 (**Figures 5A, B**), which indicate marked divergence in transcriptional regulatory programs between cDCs subsets under septic conditions. KEGG enrichment analysis of regulon target genes revealed that NFKB1 target genes were significantly enriched in the ubiquitin-mediated proteolysis pathway (**Figure 5C**), suggesting a central role for NFKB1 in coordinating ubiquitination-related transcriptional programs in cDC1. Ubiquitination-related NFKB1 target genes were then used to construct a PPI network using the STRING database. The resulting network exhibited dense connectivity, with TRAF6 and UBE2N emerging as central nodes (**Figure 5D**). To further prioritize sepsis-relevant URGs within this regulatory framework, we applied three complementary machine-learning feature-selection approaches. LASSO regression with ten-fold cross-validation identified 12 genes with non-zero coefficients at the optimal penalty parameter (**Figures 5E, F**). A random forest model ranked genes by MeanDecreaseGini and retained seven genes with importance values above the median (**Figure 5G**). SVM-RFE identified an optimal feature set comprising eight genes (**Figure 5H**). Importantly, these machine-learning approaches were applied independently and without prior feature weighting, allowing for unbiased cross-method comparison. Intersecting the results of all three machine-learning approaches yielded seven consensus hub genes: BIRC3, CUL1, TRAF6, UBE2F, UBE2N, UBE2R2 and UBE3A (**Figure 5I**). These genes represent high-confidence ubiquitination-related candidates supported by convergent evidence from transcriptional regulation, network topology, and multi-algorithm feature selection.

### 3.5 Single-cell and multi-cohort validation identify four ubiquitination-related genes with robust diagnostic relevance in sepsis

**Figure 6:**
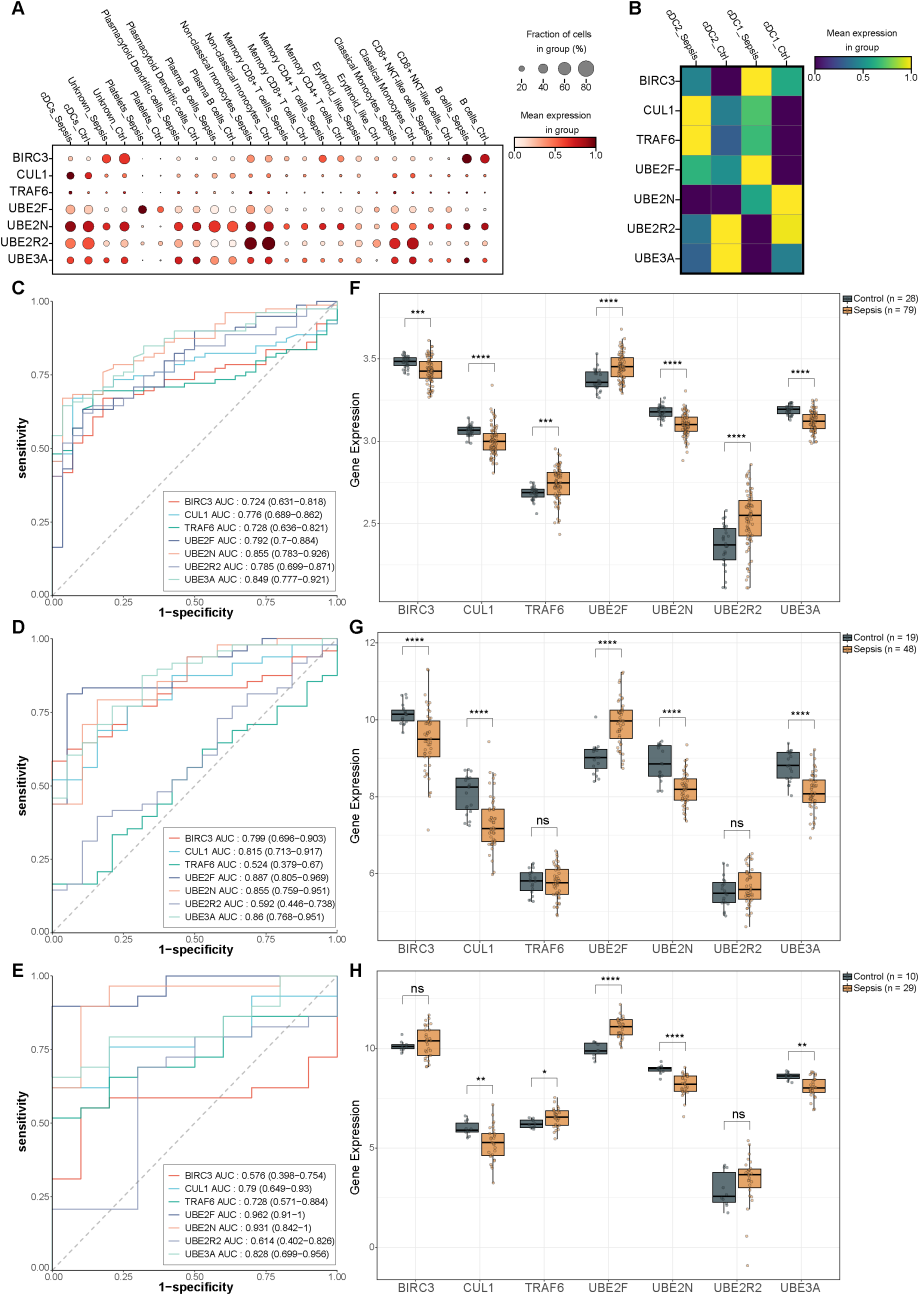
Validation of ubiquitin−related hub genes: single−cell expression and multi−cohort ROC/boxplot analyses. Datasets used in panels C–E and F–H correspond to GSE69528, GSE13015_GPL6106, and GSE13015_GPL6947, respectively. **(A)** Dot plot showing expression of key genes (BIRC3, CUL1, TRAF6, UBE2F, UBE2N, UBE2R2, UBE3A) across major cell populations. Dot color denotes average expression (scaled 0.0–1.0); dot size indicates fraction of cells expressing the gene (20%–80%). **(B)** Dot plot of the same key genes across classical dendritic cell subgroups (cDC1_Ctrl, cDC1_Sepsis, cDC2_Ctrl, cDC2_Sepsis). **(C–E)** ROC curves for hub genes in independent bulk cohorts (AUC and 95% CI reported). **(F–H)** Boxplots comparing expression of key genes between sepsis patients and healthy controls across three independent cohorts. Gray boxes denote controls; orange boxes denote sepsis. Sample sizes are indicated in legends. Statistical comparisons were performed using the Mann–Whitney U test. Significance annotations: ****P < 0.0001; **P < 0.01; *P < 0.05; ns, not significant (P > 0.05). Cohort sample sizes: F (n = 28 vs. 79), G (n = 19 vs. 48), H (n = 10 vs. 29).

We next assessed whether the prioritized ubiquitination-related candidates show reproducible diagnostic value across independent cohorts. In scRNA-seq data, dot plot analysis across all immune cell populations revealed that the seven candidate genes were highly expressed in cDCs, non-classical monocytes and B cells (**Figure 6A**). Within cDCs subsets, both the proportion of expressing cells and the average expression levels of these genes differed markedly in cDC1 from septic samples compared with cDC1 controls and cDC2 populations, indicating aberrant activation of ubiquitination-related programs specifically in cDC1 during sepsis (**Figure 6B**). To assess the diagnostic potential of these candidate genes, we performed ROC analysis across three independent bulk transcriptomic cohorts (GSE69528, GSE13015_GPL6106 and GSE13015_GPL6947). Genes achieving an AUC greater than 0.7 were considered to have satisfactory discriminative performance. ROC analysis demonstrated that CUL1, UBE2F, UBE2N and UBE3A consistently exhibited AUC values exceeding 0.7 across all three cohorts, supporting their robust diagnostic relevance (**Figures 6C–E**). In contrast, TRAF6 and UBE2R2 showed reduced performance in GSE13015_GPL6106 (AUC = 0.576 [0.398–0.754] and 0.614 [0.402–0.826], respectively), while BIRC3 and UBE2R2 exhibited suboptimal discrimination in GSE13015_GPL6947 (AUC = 0.524 [0.379–0.670] and 0.592 [0.446–0.738], respectively), indicating cohort-dependent variability for these genes. Expression distinctions were further explored through boxplots in all cohorts, revealing consistent downregulation of CUL1, UBE2N, and UBE3A in sepsis compared to controls, while UBE2F exhibited robust and significantly elevated expression across all datasets (P < 0.05) (**Figures 6F–H**). In conclusion, these results identify CUL1, UBE2F, UBE2N and UBE3A as the most consistently sepsis-associated ubiquitination-related genes, with strong and reproducible diagnostic potential.

### 3.6 UBE2F modulates sepsis progression by regulating DCs activation, inflammatory responses and cell fate

**Figure 7:**
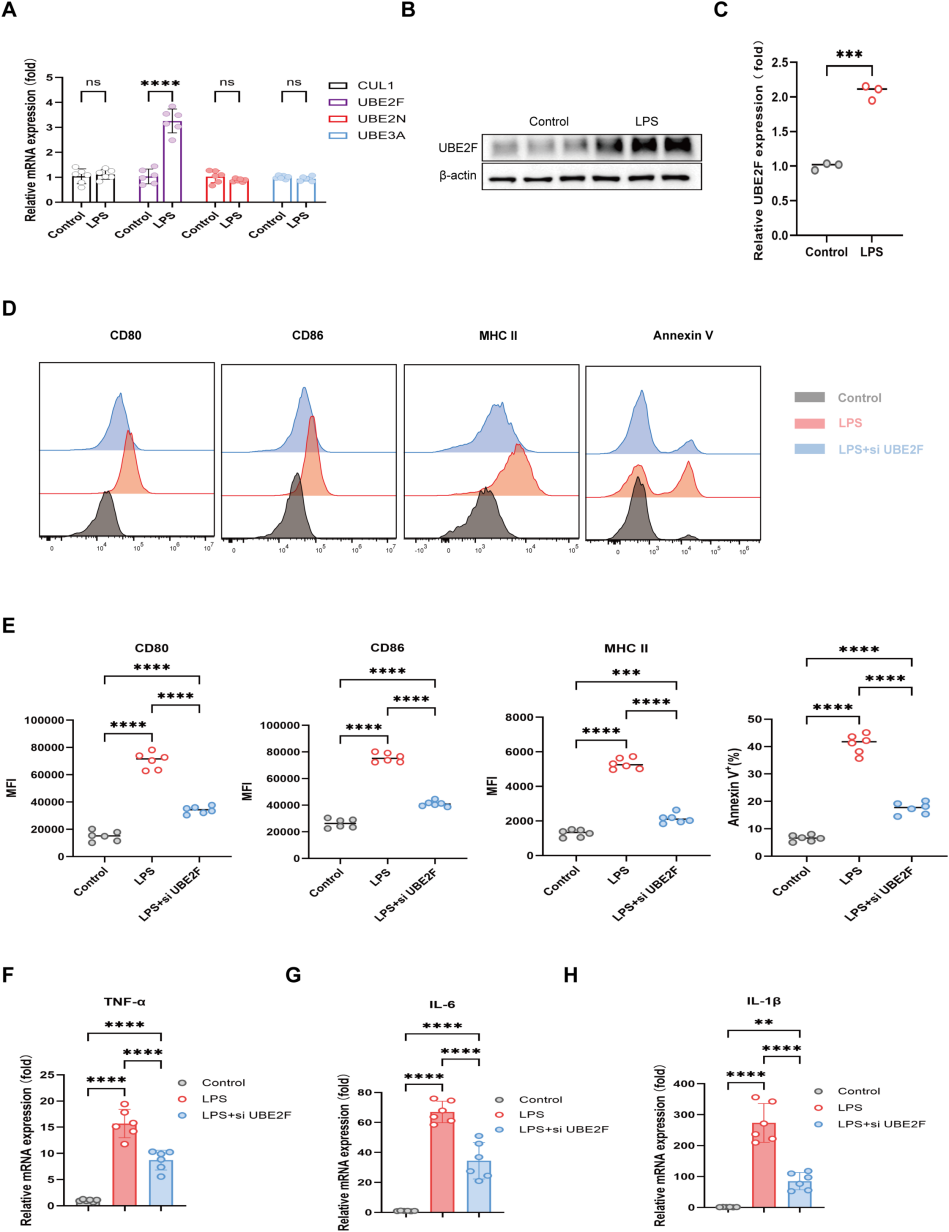
Validation of key ubiquitination-related genes and functional characterization of UBE2F in a DCs sepsis model. **(A)** mRNA expression validation of four core ubiquitination-related genes in LPS-stimulated DCs. **(B, C)** Representative Western blots **(B)** and quantification **(C)** of UBE2F protein levels in control versus LPS-treated DCs. **(D, E)** Effect of UBE2F knockdown on DCs maturation and apoptosis, visualized by flow cytometry histograms **(D)** and statistical summaries **(E)**. **(F–H)** Regulation of pro-inflammatory cytokines by UBE2F. qRT-PCR analysis of TNF-α **(F)**, IL-6 **(G)**, and IL-1β **(H)** mRNA levels. Data are presented as mean ± SEM (n = 6 per group), Statistical significance was determined using one-way ANOVA followed by Tukey’s *post hoc* test, or Log-rank (Mantel-Cox) test for survival analysis. ns, not significant; *P < 0.05; **P < 0.01; ***P < 0.001; ****P < 0.0001.

DC2.4, a dendritic cell line, was cultured and stimulated with LPS to establish an *in vitro* sepsis-like inflammatory model (44). qRT-PCR analysis demonstrated that LPS stimulation significantly increased the mRNA expression of UBE2F, while CUL1, UBE2N, and UBE3A showed no significant changes (**Figure 7A**). Consistent with the transcriptional upregulation observed by qPCR, we further assessed UBE2F expression at the protein level. Western blot (**Figures 7B, C**) and immunofluorescence analyses (**Supplementary Figure 2B**) demonstrated that UBE2F protein expression was significantly increased in DCs following LPS stimulation. To investigate the functional consequences of UBE2F upregulation, UBE2F expression was silenced in DCs using siRNA (**Supplementary Figure 2A**). Flow cytometric analysis revealed that LPS stimulation induced robust upregulation of maturation markers CD80, CD86 and MHC class II, accompanied by increased apoptosis. Notably, UBE2F knockdown significantly attenuated LPS-induced DCs maturation and reduced apoptotic cell death (**Figures 7D, E**). Furthermore, LPS-triggered expression of proinflammatory cytokines, including TNF-α, IL-1β and IL-6, was markedly suppressed following UBE2F silencing (**Figures 7F–H**).

**Figure 8:**
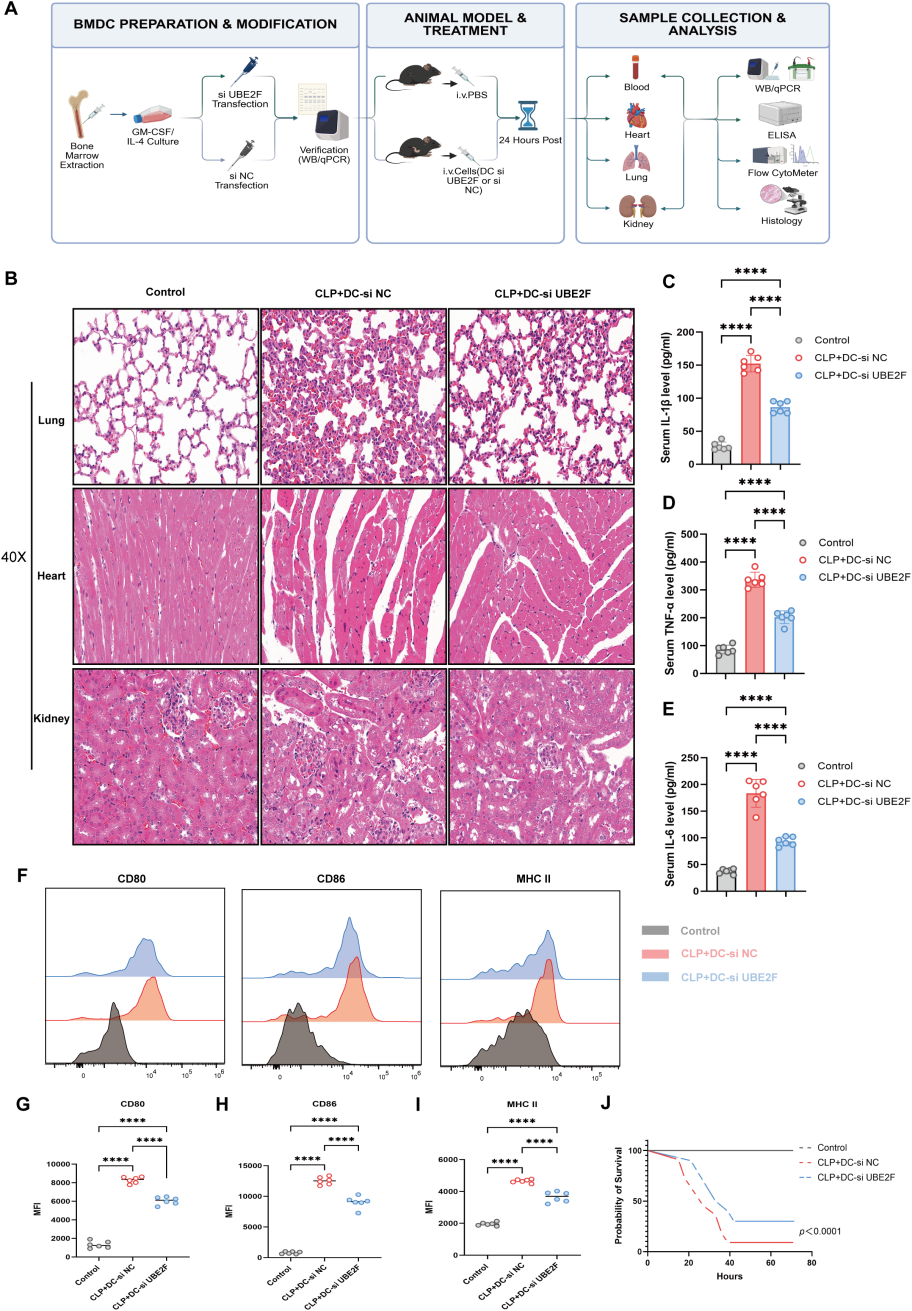
*In vivo* validation of UBE2F knockdown in alleviating sepsis progression in mice. **(A)** Schematic illustration of the CLP mouse model establishment and the adoptive transfer protocol for UBE2F-silenced BMDCs. **(B)** Representative H&E staining showing pathological changes in lung, heart, and kidney tissues. **(C–E)** Serum concentrations of pro-inflammatory cytokines IL-1β **(C)**, TNF-α **(D)**, and IL-6 **(E)** quantified by ELISA. **(F–I)** *In vivo* assessment of cDCs maturation. Representative flow cytometry histograms **(F)** and statistical quantification of MFI for CD80 **(G)**, CD86 **(H)**, and MHC II **(I)**. **(J)** Kaplan-Meier survival analysis of septic mice following indicated treatments. Data are presented as mean ± SEM (n = 6 mice per group). Statistical significance was determined using one-way ANOVA followed by Tukey’s *post hoc* test, or Log-rank (Mantel-Cox) test for survival analysis. ns, not significant; *P < 0.05; **P < 0.01; ***P < 0.001; ****P < 0.0001.

*In vivo*, high-purity BMDCs were generated by *in vitro* differentiation of bone marrow cells (**Supplementary Figure 2C**). A CLP mouse model of sepsis was then established, and UBE2F-knockdown BMDCs were intravenously administered to reduce UBE2F expression (**Figure 8A**). H&E staining demonstrated that administration of UBE2F-silenced DCs markedly alleviated sepsis-associated tissue injury in the lung, heart and kidney (**Figure 8B**). In line with the *in vitro* findings, *in vivo* UBE2F knockdown significantly reduced the expression of maturation markers CD80, CD86, and MHC class II in splenic cDCs, as determined by flow cytometry (**Figures 8F–I** and **Supplementary Figure 2D**), as well as serum levels of TNF-α, IL-1β, and IL-6 (**Figures 8C–E**). Importantly, Kaplan–Meier survival curves analysis revealed a significant reduction in mortality among septic mice receiving UBE2F-knockdown DCs (**Figure 8J**). Taken together, these *in vitro* and *in vivo* findings demonstrate that UBE2F plays a critical role in sepsis progression by regulating DCs activation, inflammatory responses and cell fate.

## 4 Discussion

Sepsis remains a leading cause of mortality worldwide, owing to its complex and heterogeneous pathophysiology characterized by dysregulated immune activation, aberrant inflammatory signaling and widespread organ dysfunction (3). Although increasing evidence implicates ubiquitination in sepsis-associated organ injury (45), prior studies have largely focused on isolated genes in bulk tissues, failing to resolve the specific immune cell subsets that drive ubiquitination-mediated pathology. Consequently, the regulatory logic linking intercellular signaling cues to intracellular ubiquitination networks remains a “black box”. In the present study, through integrative single-cell and bulk transcriptomic analyses combined with functional validation, we identify cDC1 as a central ubiquitination-centric immune cell population in sepsis and delineate a regulatory framework linking ubiquitination, TNF signaling and cDCs activation. Moreover, we identify four ubiquitination-related genes—CUL1, UBE2F, UBE2N and UBE3A—as key molecular components associated with sepsis progression, and provide *in vivo* and *in vitro* evidence supporting a pivotal role for UBE2F. This work provides mechanistic insight into immune reprogramming in sepsis and highlights the therapeutic potential of targeting the cDC1–ubiquitin axis.

By analyzing over 315,000 single cells, cDC1 showed the highest transcriptional sensitivity to septic perturbation and the most pronounced activation of ubiquitination-related gene programs. cDCs are professional antigen-presenting cells derived from bone marrow hematopoietic progenitors and are commonly divided into cDC1 and cDC2 subsets based on developmental transcription factors, surface markers and functional specialization (46, 47). cDC1 is involved in multiple biological processes, including atherosclerosis (48), cancer immunotherapy (49), and renal tissue homeostasis (50). In sepsis, cDC1 displayed enhanced antigen processing and presentation activity despite a reduced abundance, suggesting dysregulated antigen presentation that may contribute to immune imbalance. Our CellChat analysis suggests that cDC1 may function as an important communication hub within the septic myeloid compartment. Rather than serving only as professional antigen-presenting cells, cDC1 appear to participate actively in shaping inflammatory signal propagation among innate immune cells. In particular, the enrichment of TNF-centered communication involving cDC1 points to a role for this subset in coordinating myeloid cell responses during septic immune remodeling. TNF is a central driver of systemic inflammation in sepsis, and excessive TNF signaling can exacerbate organ injury and mortality (1). The predominance of the TNF–TNFRSF1B interaction is also noteworthy, as TNFRSF1B is more closely linked to immune regulation and cell activation than to the broader cytotoxic responses classically associated with TNFRSF1A. This pattern may therefore reflect a more structured immunoregulatory circuit rather than nonspecific inflammatory amplification. These observations are consistent with our finding that septic cDC1 exhibit enhanced ubiquitination-related activity. Given the well-established role of ubiquitination in regulating TNF receptor signaling, including downstream NF-κB activation and signal complex stability, it is plausible that altered ubiquitination in cDC1 contributes to their rewired communication behavior in sepsis. Taken together, these data support a model in which cDC1 act as a functionally reprogrammed myeloid subset that links dysregulated ubiquitination to TNF-dependent immune network remodeling. However, the mechanistic basis of this interaction and its causal contribution to sepsis progression will require further experimental validation.

Through integration of bulk RNA-seq data, weighted gene co-expression network analysis and transcription factor inference, we further delineated ubiquitination-related regulatory modules associated with sepsis. NFKB1 emerged as a central transcriptional regulator in cDC1, with its target genes significantly enriched in ubiquitin-mediated proteolysis pathways, underscoring the tight coupling between inflammatory signaling and ubiquitination machinery. Multi-cohort validation and machine-learning prioritization then converged on four ubiquitination-related genes (CUL1, UBE2F, UBE2N and UBE3A) with reproducible diagnostic performance. These genes occupy distinct yet interconnected positions within the ubiquitin–proteasome system. CUL1 functions as a scaffold component of the SCF E3 ubiquitin ligase complex, orchestrating substrate ubiquitination and proteasomal degradation (51–53). UBE2N mediates Lys63-linked polyubiquitination, a non-degradative modification critical for signal transduction in innate immunity and inflammatory homeostasis (54–56). UBE3A, a HECT-type E3 ligase, regulates protein stability and cellular function through targeted ubiquitination and has been implicated in neural and glial biology (57, 58). Collectively, the functional attributes of these genes support their potential involvement in sepsis-associated immune dysregulation and intercellular communication.

Among these candidates, UBE2F emerged as the most consistently upregulated gene across datasets and exhibited the strongest functional relevance in experimental models. UBE2F is a core E2 conjugating enzyme in the non-canonical ubiquitin-like modification pathway, facilitating NEDD8 conjugation and downstream signaling (59). Aberrant upregulation of UBE2F is associated with hyperactivation of non-canonical ubiquitination pathways (60), and it is also involved in the progression of lung, pancreatic and liver cancers (61–63). In our study, given the extreme scarcity of primary cDC1 cells in peripheral blood, BMDCs and a DCs line were employed for functional validation to ensure experimental robustness. Our data suggest that UBE2F silencing reduced LPS-induced DCs maturation, cytokine production and apoptosis *in vitro*, and alleviated multi-organ injury, suppressed systemic inflammation and improved survival in a CLP sepsis model *in vivo*. The concordance between *in vitro* and *in vivo* data supports a causal role for UBE2F in regulating DCs activation and fate during sepsis progression.

Several limitations of this study should be acknowledged. First, clinical validation was primarily based on publicly available transcriptomic datasets, and multicenter, prospective cohorts are required to further assess associations between core gene expression, disease severity indices (such as SOFA score), and clinical outcomes. Second, some public datasets had relatively limited sample sizes, which may affect statistical robustness and generalizability. Third, because primary cDC1 cells are extremely scarce in peripheral blood and tissues, the functional role of UBE2F was investigated mainly in DC2.4 cells and GM-CSF/IL-4-derived BMDCs. Although these systems are widely used and technically practical for assessing dendritic cell activation, apoptosis, and inflammatory cytokine production, they do not fully recapitulate the phenotype and functional specialization of endogenous cDC1. In particular, GM-CSF-derived BMDCs are generally considered closer to inflammatory monocyte-derived dendritic cells than to bona fide steady-state cDC1. As a result, our experimental data support a role for UBE2F in dendritic cell activation and innate inflammatory responses during sepsis, but the extent to which these findings reflect cDC1-specific functions—especially specialized processes such as antigen cross-presentation and CD8^+ T-cell priming—remains to be established. Future studies using Flt3L-driven cultures, purified primary cDC1, or cDC1-specific conditional knockout models such as Xcr1-Cre or Clec9a-Cre mice will be important for defining the *in vivo* role of the UBE2F–cDC1 axis more precisely. Addressing these limitations will be essential for translating ubiquitination-targeted strategies into clinical applications.

## 5 Conclusion

By integrating single-cell and bulk transcriptomic analyses with network biology, machine learning and functional validation, this study identifies cDC1 as a central immune population exhibiting aberrant ubiquitination activation in sepsis. We further implicate TNF signaling and an NFKB1-centered transcriptional network as key drivers of ubiquitination dysregulation. Additionally, we identify four ubiquitination-related genes (CUL1, UBE2F, UBE2N, and UBE3A) that show consistent diagnostic relevance in sepsis. Among these candidates, UBE2F emerges as the most functionally relevant gene, as functional experiments demonstrate its critical role in regulating dendritic cell activation and sepsis progression.

## Data Availability

The datasets presented in this study can be found in online repositories. The names of the repository/repositories and accession number(s) can be found in the article/**Supplementary Material**.
