# Supplementary material for: Integrated single-cell and bulk transcriptomic analyses reveal cDC1-centered ubiquitination dysregulation and identify UBE2F as a critical regulator in sepsis
Source: Front Immunol. 2026 Apr 22;17:1805849. doi: 10.3389/fimmu.2026.1805849 (PMC13143726; doi:10.3389/fimmu.2026.1805849)
Supplement: Supplementary file 3 [file DataSheet3.docx]

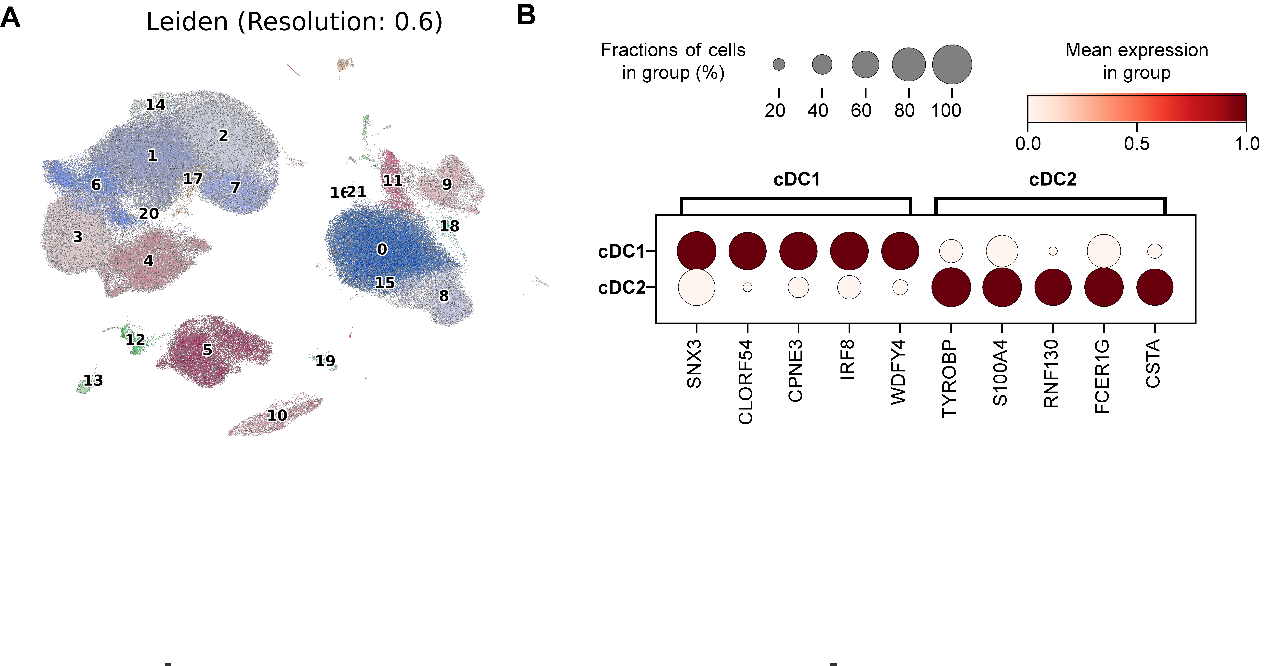


Figure S1. Characterization of single-cell clusters and Marker gene expression maps for cDCs subsets. **A**. UMAP visualization of cell clusters identified via Leiden clustering (resolution: 0.6). **B**. Marker gene expression maps for cDC1 and cDC2, illustrating canonical markers distinguishing the two subsets.


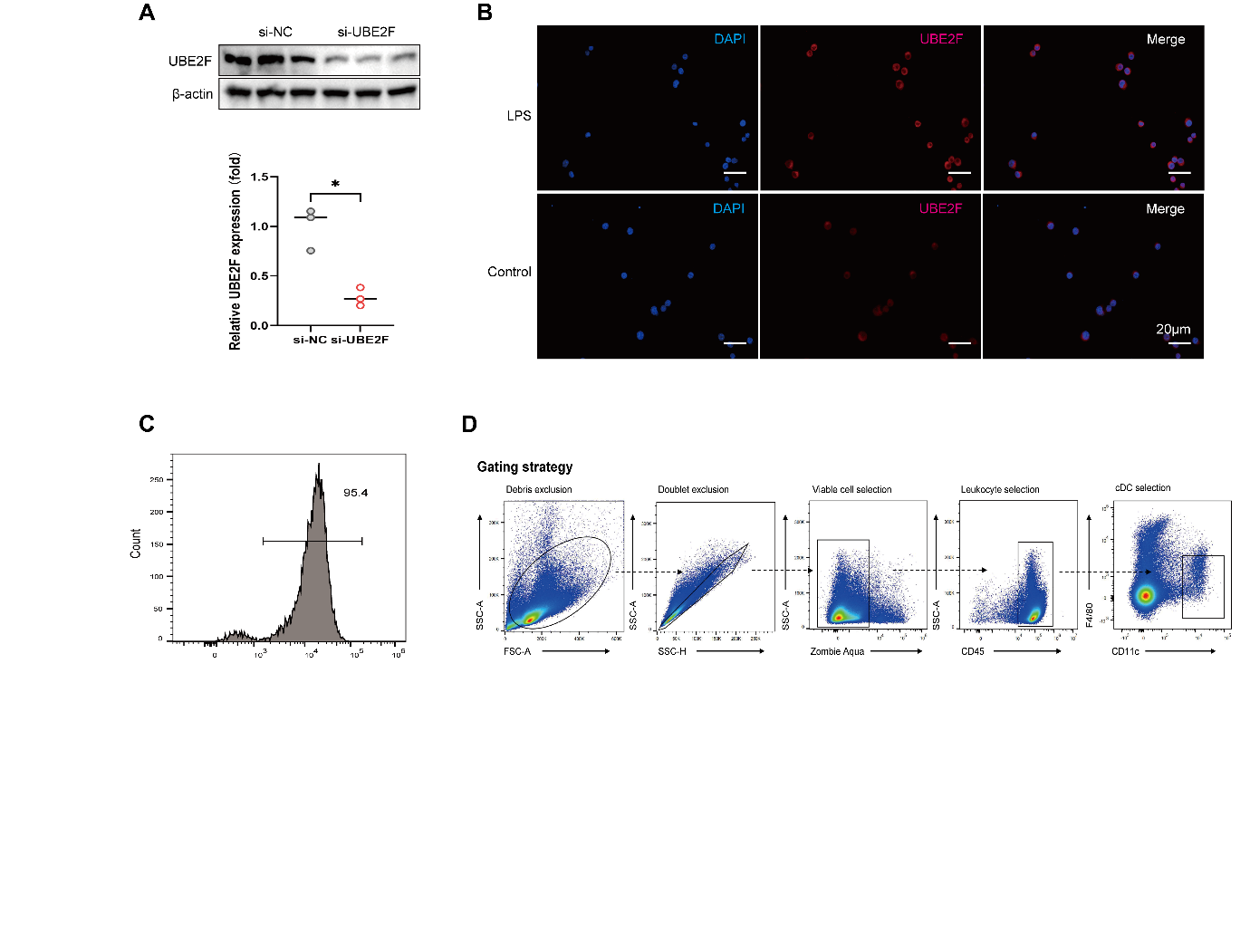


Figure S2. Validation of dendritic cell models and flow cytometric gating strategy. **A**. Western blot analysis confirming the knockdown efficiency of UBE2F in the DC2.4 cell line. **B**. Immunofluorescence staining showing increased expression of UBE2F in DC2.4 cells following LPS stimulation compared with control cells. Nuclei were counterstained with DAPI. Scale bar, 20 μm. **C**. Flow cytometric analysis of BMDCs to assess cell purity. **D**. Representative flow cytometric gating strategy for the identification of cDCs isolated from mouse spleens, including sequential gating for debris exclusion, doublet exclusion, viable cells, CD45⁺ leukocytes, and CD11c⁺ cDCs populations.
